# Camel Whey Protein Attenuates Acute Heat Stress-Induced Kidney Injury in Rats by Up-Regulating CYP2J Activity and Activating PI3K/AKT/eNOS to Inhibit Oxidative Stress

**DOI:** 10.3390/vetsci11110524

**Published:** 2024-10-28

**Authors:** Xiaoxia Jing, Donghua Du, Bin Hou, Deng Zhan, Surong Hasi

**Affiliations:** 1 Key Laboratory of Clinical Diagnosis and Treatment Technology in Animal Disease, Ministry of Agriculture and Rural Affairs, College of Veterinary Medicine, Inner Mongolia Agricultural University, Hohhot 010018, China; jingxx0325@126.com (X.J.);; 2 Department of Veterinary Medicine, College of Animal Science and Technology, Hebei North University, Zhangjiakou 075131, China; 3 Ordos Vocational College of Eco-Environment, Ordos 017010, China

**Keywords:** camel whey protein, natural antioxidants, oxidative stress, kidney injury

## Abstract

Camel whey protein (CWP) is a potent natural antioxidant, noted for its abundance of antioxidant amino acids. Despite its promising properties, the precise mechanisms underlying its effects remain inadequately explored. This study aims to investigate the impact of CWP on kidney damage induced by acute heat stress in rats, as well as to elucidate its mechanism of action. We assessed CWP’s influence on cytochrome P450 2J (CYP2J) activity during heat stress, measured oxidative stress levels, and evaluated renal injury using CYP2J knockout rats. Our findings indicate that acute heat stress reduces CYP2J expression, while CWP administration restores CYP2J activity and enhances PI3K/Akt signaling. However, CWP did not mitigate oxidative stress-induced kidney damage in CYP2J^−/−^ rats, suggesting the necessity of CYP2J for its protective effects.

## Simple Summary

Camel whey protein is recognized for its natural antioxidant properties, attributed to its high content of antioxidant amino acids. Oxidative stress caused by heat stress poses significant harm to the body, often leading to kidney injury. This study aimed to examine the impact of camel whey protein intervention on kidney function in rats experiencing acute heat stress, as well as to explore the underlying mechanisms involved. We assessed the activity of the cytochrome P450 2J enzyme under conditions of heat stress to evaluate oxidative stress levels and kidney damage, utilizing knockout rat models. Our findings indicated that acute heat stress led to a decrease in cytochrome P450 2J expression. However, the administration of camel whey protein significantly enhanced its activity and activated associated signaling pathways. Conversely, in knockout rats, camel whey protein did not mitigate oxidative stress-related kidney damage. This research highlights the potential of camel whey protein as a protective agent against acute heat stress-induced kidney injury and the necessity of CYP2J for its protective effects.

## 1. Introduction

The fat globules in camel milk are relatively small, and its protein structure closely resembles that of human milk, which enhances digestion and absorption. Camel milk is hypoallergenic due to the absence of beta-lactoglobulin, making it an ideal substitute for infants, young children, and individuals with allergies [1]. Camel whey protein (CWP), a protective protein in camel milk, is most concentrated in colostrum and gradually decreases over time. CWP exhibits potent antioxidant properties, functioning as a natural antioxidant due to its bioactive peptides, which scavenge free radicals and mitigate oxidative stress-related diseases [2,3]. Hua Shi and colleagues (2023) investigated camel milk collected from various times and locations, revealing that CWP composition varies with season and location [4]. Additionally, due to proteins’ susceptibility to heat denaturation, the impact of temperature and time on CWP’s nutrient content during sterilization is crucial [5]. A comparative study by Chen Qi et al. (2023) evaluated the effects of sterilization at different temperatures and durations, finding that treatment at 65 °C for 30 min minimized the impact on CWP components [6]. This approach resulted in the lowest degree of denaturation, minimal changes in physicochemical properties, and some enhancement in CWP’s emulsification and water-holding properties.

CWP contains bioactive peptides that can scavenge free radicals. Hou Zhimei et al. (2020) have suggested that CWP may provide a protective effect on pancreatic islet β-cells, potentially promoting their growth and enhancing insulin secretion [7]. Additionally, CWP appears to be a promising preventive strategy for type II diabetes, with its effects linked to anti-oxidative stress (OS) mechanisms [8]. After digestion by pepsin, CWP inhibits dipeptidyl peptidase activity, activates insulin receptors, and alleviates diabetic complications [9]. Lactoferrin, a major antioxidant component of CWP, demonstrates a total antioxidant capacity comparable to that of vitamin C. Furthermore, CWP can mitigate oxidative stress and reduce methotrexate-induced renal injury by activating the PI3K/AKT/eNOS signaling pathway [5,10].

The cytochrome P450 enzyme family represents a diverse superfamily of enzymes involved in biotransformation, with widespread distribution throughout the body. Among these, CYP2J serves as an arachidonic acid epoxidase, metabolizing arachidonic acid to produce four regioisomers of eicosatrienoic acids (EETs). EETs enhance the function of vascular smooth muscle cells by modulating calcium and potassium ion channels, leading to vasodilation and blood pressure regulation. Additionally, EETs inhibit apoptosis and promote endothelial cell proliferation by upregulating endothelial nitric oxide synthase (eNOS) expression via the activation of the phosphoinositide 3-kinase (PI3K)/AKT/eNOS signaling pathway [11,12].

Previous studies from our laboratory have demonstrated that CYP2J enzymes are most abundantly expressed in the liver and kidneys of Bactrian camels [13]. This expression pattern may be linked to the unique biological characteristics of Bactrian camels, which thrive in arid or semi-arid desert environments characterized by high temperatures year-round, yet do not exhibit symptoms of heat stress or heat stroke. Furthermore, CYP2J enzymes confer various protective effects against oxidative stress (OS). Synthesized EETs not only exhibit efficacy in ischemia-reperfusion scenarios but also possess robust antioxidant and anti-inflammatory properties. They can inhibit the activation of inflammatory cells and the release of pro-inflammatory mediators, thereby mitigating OS-induced cellular damage [14,15,16]. Additionally, CYP2J-mediated epoxy lipids can activate the expression of cellular detoxification enzyme systems by inducing the nuclear factor erythroid 2-related factor 2 (Nrf2), enhancing the body’s resilience to oxidative stress [17].

As global temperatures rise, the incidence of heat stress (HS) is increasing. Exposure to heat stress can cause varying degrees of damage to tissues and organs, with the liver and kidneys being particularly vulnerable [18]. Enhancing antioxidant capacity is an effective strategy to mitigate HS-induced tissue damage. Previous studies conducted in our laboratory have shown that CWP alleviates HS-induced apoptosis and damage in rat hepatocytes by activating antioxidant pathways, demonstrating significant efficacy [19]. Additionally, hydrolyzed CWP has been found to mitigate HS-induced hepatocyte damage in rats [20]. Based on these findings, it can be concluded that CWP plays a crucial role in reducing HS-induced tissue damage. Therefore, the current study aims to further investigate the protective effects of CWP on renal tissue, to confirm these effects, and to elucidate the underlying molecular mechanisms using gene knockout technology.

## 2. Materials and Methods

### 2.1. Chemicals and Antibodies

Trizma Hydrochloride Solution (Merck KGaA, Darmstadt, Germany-No. T2663); Proteinase K (Merck KGaA, Darmstadt, Germany-No. MK539480); Sp6 In Vitro Transcription Kit (Thermo Fisher Scientific, Shanghai, China-AM1310); Dynabeads™ RNA Purification Kit (Thermo Fisher Scientific, Shanghai, China-65032D); Universal Genomic DNA Extraction Kit (TaKaRa MiniBEST, Beijing, China-9765); 2 × Taq Master Mix (Dye Plus) (Vazyme, Nanjing, China-P131); DNA Marker (Thermo Scientific GeneRuler 1 kb DNA Marker (Thermo Scientific GeneRuler 1 kb DNA Ladder #SM0311), Protein Quantification BCA Assay Kit, and cell lysate were purchased from Thermo Fisher Scientific, Shanghai, China. Cytochrome P450 subenzyme CYP2J activity fluorescence quantitative detection kit (GENMED SCIENTIFICS INC., South San Francisco, CA, USA-GMS18190.2 v.A); Bovine whey protein (BWP) was purchased from Hilmar, TX, USA. β-actin antibody (ab8227) was purchased from Abcam; PI3K-P110 (AF5112), P-AKT (Ser473) (AF0016), AKT (AF6261), P-eNOS (Ser1177) (AF3247), and eNOS (AF0096) were purchased from Affinity Biosciences; sheep anti-rabbit IgG(H + L)-HRP (LK2001) was purchased from the Tianjin Three Arrows Biotechnology Co. (Tianjin, China).

### 2.2. Ethics Statement and Animals

Six-week-old Specific Pathogen Free healthy SD rats. Half males and half females, divided into seven groups of six weighing 220 ± 20 g, were randomly housed in cages with free access to food and water, in an environment with a controlled temperature of 25 ± 2 °C and humidity of 50 ± 5%. A 12 h light-dark cycle was applied and the animals were acclimatized for 10 days. The experiment was approved by the Scientific Research and Ethics Committee of Inner Mongolia Agricultural University (GB/T 35892-2018).

### 2.3. Preparation of Camel Whey Protein

The fresh Bactrian camel milk was collected aseptically from at least 20 healthy camels of the local farm in Alashan, Inner Mongolia, China. It was then centrifuged to remove the milk fat layer. The pasteurized milk was purified with ammonium sulphate and dialyzed overnight at 4 °C using dialysis bags with a molecular weight cut-off of 3500. The dialyzed CWP was pre-cooled at −80 °C for 24 h and then lyophilized in a freezer at −50 °C, 2–10 Pa. The final CWP powder was collected and stored at −20 °C. The final CWP powder was then stored in a freezer at −20 °C [21,22,23].

### 2.4. Construction of CYP2J^−/−^ Knockout Rats

The complete gene sequence of rat CYP2J3 (ID: 313375) was retrieved from the NCBI database. The sequence with the lowest off-target analysis score was selected as the optimal target for editing. The gRNAs were synthesized and purified by SAIYE Bioscience Ltd. (Guangdong, China), and stored at −80 °C. The sequences of the gRNAs used were:

gRNA-A1: TGCAGCTACCCAATGTCACTAGG

gRNA-A2: AACTTGTAGTTCTAATATGCTGG

Cas9 mRNA was generated using a Cas9 plasmid as the template, following the instructions provided in the SP6 in vitro transcription kit. The mRNA was subsequently purified with an RNA purification kit and stored at −80 °C.

Seven-week-old healthy SD male rats were subjected to vasectomy, and pseudo-pregnant females were prepared through abdominal incision (indicated by the formation of a negative embolus the following day) [24]. Fertilized eggs were collected, transferred into M2 solution without hyaluronidase, and incubated at 37 °C for 2 h. The preserved gRNA and Cas9 mRNA were then co-injected into the fertilized eggs, which were incubated at 37 °C for 30 min. Morphologically intact eggs were selected and transplanted into pseudo-pregnant females for natural implantation.

DNA was extracted for PCR genotyping using the following primers, which had an annealing temperature of 60.0 °C:

Forward primer (F1): 5′-CCAGAACTTGTCCGTCACTACTA-3′

Reverse primer (R1): 5′-AGACGGCTGACAGTGTTGAATTA-3′

Targeted allele: 465 bp

Wildtype allele: 11724 bp

PCR products were purified and verified by sequencing, with sequencing primers as follows:

Sequence primer (F1): 5′-CCAGAACTTGTCCGTCACTACTA-3′

Progenies were produced by crossbreeding heterozygous target mice to generate homozygous target mice. The genotypes were determined by PCR following the birth of the offspring, which occurred 5–7 days post tail clipping. The validation primers, designed and synthesized by SAIYE Bioscience Ltd., were:

PCR Primers 1 (Product size: 465 bp, Wildtype allele: 11724 bp):

F1: 5′-CCAGAACTTGTCCGTCACTACTA-3′

R1: 5′-AGACGGCTGACAGTGTTGAATTA-3′

PCR Primers 2 (Product size: 822 bp):

F2: 5′-TAACTTTAACATCACTTCGCTGCC-3′

R1: 5′-AGACGGCTGACAGTGTTGAATTA-3′

### 2.5. Experimental Design and Treatment Protocol

Six-week-old rats were acclimatized in the laboratory for 1 week and then randomly assigned to one of seven groups [25,26]:

Control Group: Received saline via gavage at a dose of 1 mL/day for 10 days.

Model Group: Received saline via gavage at a dose of 1 mL/day for 10 days, followed by acute heat stress (temperature: 40 ± 0.5 °C; humidity: 60 ± 5%; duration: 3 h).

CWP Groups: Divided into low, medium, and high dose groups, receiving CWP at doses of 100, 200, and 400 mg/kg per day, respectively, for 10 days. Acute heat stress was then induced.

Bovine whey protein (BWP) Group: Received BWP at a dose of 400 mg/kg per day for 10 days, followed by acute heat stress.

NAC Positive Control Group: Received N-acetylcysteine (NAC) at a dose of 100 mg/kg per day via intraperitoneal injection for 10 days, followed by acute heat stress.

Following 3 h of heat stress, the rats were returned to a room temperature environment for recovery. After 9 h of rewarming, they were anesthetized, and blood samples were collected from the heart. Kidney tissue was then harvested: a portion was fixed in 4% paraformaldehyde for pathological section analysis, while the remaining tissue was stored at −80 °C for subsequent experiments.

### 2.6. Assessment of the Nephrotoxicity Markers (BUN, S-Cr and NGAL)

Serum samples were centrifuged at 3000 rpm for 10 min and analyzed according to the kit instructions. Serum creatinine (S-Cr) absorbance was measured at 546 nm, blood urea nitrogen (BUN) at 640 nm, and neutrophil gelatinase-associated lipocalin (NGAL) at 450 nm. Concentrations were determined by fitting a logistic four-parameter curve using ELISAcalc (v 0.2).

### 2.7. Histopathology of Kidney Tissues

Kidney tissue blocks were fixed in 4% paraformaldehyde overnight at room temperature. After fixation, the tissues were processed, embedded in paraffin, and sectioned into 4 μm slices using a microtome. The sections were then dewaxed, rehydrated, stained with hematoxylin and eosin, and mounted with neutral gum. Images were captured using a standard light microscope.

### 2.8. Oxidative Stress Markers (SOD, MDA, GSH, GSH-PX, CAT, T-AOC)

Superoxide dismutase (SOD) activity was measured using the water-soluble tetrazolium salt-1 assay, while malondialdehyde (MDA) levels were assessed via the thiobarbituric acid (TBA) method. Reduced glutathione (GSH) and glutathione peroxidase (GSH-PX) levels were determined using a microplate assay, and catalase activity was quantified by the ammonium molybdate method. Total antioxidant capacity (T-AOC) was measured using the ferric reducing antioxidant power (FRAP) assay. All procedures followed the standard experimental protocols.

### 2.9. Immunohistochemistry of Renal NGAL

Kidney tissues were sectioned and subjected to antigen retrieval using citric acid (pH 6.0), followed by incubation in 3% BSA at room temperature for 30 min. The sections were then incubated overnight at 4 °C with the primary antibody against NGAL (1:100) in a humidified chamber. Following this, the sections were treated with a horseradish peroxidase (HRP)-conjugated goat anti-rabbit secondary antibody (1:200) for 50 min at room temperature. Color development was achieved using diaminobenzidine (DAB), after which the slices were mounted with a sealing gel and examined under a light microscope for result interpretation.

### 2.10. TUNEL Detection of Apoptosis in Kidney Cells

Kidney tissue sections underwent proteinase K treatment for antigen retrieval, and cell nuclei were counterstained with DAPI. The sections were incubated in the dark at room temperature for 10 min, then mounted with an anti-fade mounting medium. Fluorescence microscopy was employed to visualize the sections, and images were captured. DAPI was excited with a UV light (330–380 nm) and emitted a blue light (420 nm), while CY3 was excited with a green light (510–561 nm) and emitted a red light (590 nm).

### 2.11. CYP2J Activity Assay

Serum samples were prepared by collecting whole blood from rats and processing it according to the kit’s protocol. The assay was conducted using a fluorescence spectrophotometer set at 37 °C, with an excitation wavelength of 370 nm, an emission wavelength of 450 nm, and a gain multiplier of 100.

### 2.12. ELISA Assay for Eicosatrienoic Acids Activity

Serum samples were prepared by collecting whole blood from rats and allowing the kit components to equilibrate at room temperature for at least 30 min. Standard, blank, and sample wells were prepared according to the provided instructions. The plate was then sealed with a sealing film and incubated at 37 °C for 60 min. Following incubation, the liquid was removed, and the plate was wrapped in absorbent paper and patted dry. Each well was subsequently filled with washing solution, left for one minute, and then the washing solution was removed and the plate was patted dry again. This washing procedure was repeated five times. Afterward, substrate solution was added to each well, and the plate was incubated at 37 °C for 15 min. Termination solution was then added to each well, and the OD values were measured at 450 nm within 15 min.

### 2.13. Immunoblot Analysis of PI3K/Akt/eNOS Pathway

A total of 500 μL of lysate containing protease and phosphatase inhibitors (5 mg/mL) was added to kidney tissue samples. Tissue fragmentation and lysis were achieved using a tissue homogenizer. The protein concentration was determined using the BCA assay, and an aliquot of protein (2 mg/mL) was separated by SDS-PAGE and transferred to a PVDF membrane, which was then blocked with 5% skimmed milk powder. The membrane was incubated overnight at 4 °C with primary antibodies against β-actin, PI3K-P110 (rabbit), phosphorylated-AKT (Ser473) (rabbit), Akt (rabbit), phosphorylated-eNOS (Ser1177) (rabbit), and eNOS (rabbit). Following primary antibody incubation, the membrane was probed with HRP-conjugated goat anti-rabbit secondary antibody at room temperature for 1 h. Protein bands were visualized using the ECL dual-sensitive chemiluminescence reagent, and images were captured for analysis.

### 2.14. Statistical Analysis

Experimental data were analyzed using SPSS 26.0 software, employing independent sample *t*-tests and one-way ANOVA. Data are expressed as mean ± SEM. ELISA results were calculated using ELISAcalc (v 0.2) with logistic curve fitting (four-parameter). Western blot images were processed using Adobe Photoshop CC2019, band quantification was performed with ImageJ (2.14.0), and data visualization was accomplished using GraphPad Prism 10.

## 3. Results

### 3.1. Composition of CWP

Table 1 demonstrates that albumin and α-lactalbumin are the most abundant proteins, with lactoferrin also present in notable amounts. Mass spectrometry analysis revealed that these proteins are more prevalent in CWP compared to BWP, suggesting that CWP might possess superior antioxidant capacity.

### 3.2. Effect of CWP on CYP2J and EETs Activity

**Figure 1:**
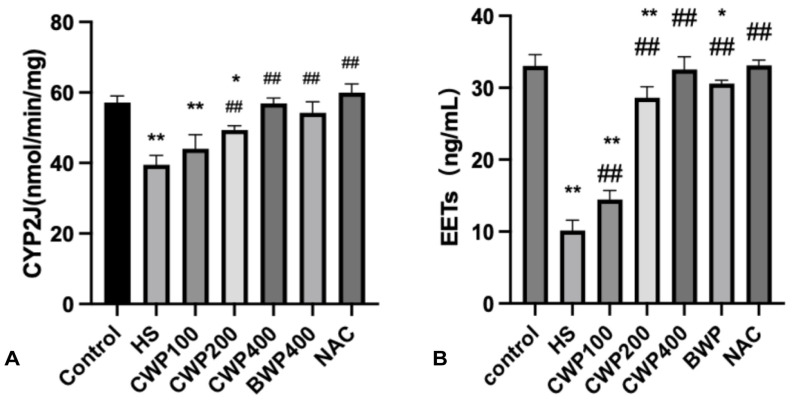
Acute heat stress inhibits CYP2J activity and the production of EETs in rats. (**A**) control group (saline gavage, 1 mL/day for 10 days), HS group (saline gavage, 1 mL/day for 10 days), CWP100 group (CWP gavage, 100 mg/kg for 10 days), CWP200 group (CWP gavage, 200 mg/kg for 10 days), CWP400 group (CWP gavage, 400 mg/kg for 10 days), BWP400 group (BWP gavage, 400 mg/kg for 10 days), and NAC group (intraperitoneal injection of N-acetylcysteine, 100 mg/kg for 10 days). Except for the control group, the other six groups underwent acute heat stress treatment for 3 h, followed by a rewarming period of 9 h, after which CYP2J activity was measured. (**B**) Similar to panel A, the groups were subjected to the same treatment regimen, and EETs levels were assessed following the 9 h rewarming period. Data are expressed as mean ± SEM, based on six animals per group across the seven groups. “*” indicates a significant difference compared to the control group (*p* < 0.05), and “**” indicates a highly significant difference compared to the control group (*p* < 0.01); and “##” indicates a highly significant difference compared with the HS group (*p* < 0.01).

Figure 1A illustrates that HS inhibits CYP2J expression in rat serum. However, with increasing doses of CWP, CYP2J expression gradually approaches normal levels observed in the control group. The CWP400 high-dose group, BWP group, and NAC-positive drug control group showed no significant difference from the control group, indicating stable CYP2J activity.

As is shown in Figure 1B, HS negatively impacts CYP2J activity and EET production. After HS, EET levels in the HS model group were significantly reduced. CWP administration mitigated this effect, with no significant difference in EET content between the CWP400 high-dose group and the NAC-positive drug control group compared to the control group. This supports the role of CWP in modulating the CYP2J-EETs system under HS conditions.

### 3.3. Gene Editing and Detection of F1 Generation Genotypes

The rat CYP2J3 gene (Gene ID: 313375; GenBank: NM_175766.3) is located on chromosome 5, spanning nine exons. Target sites for gene editing were selected within Exons 2 to 6. Genotypic PCR of F1 generation tail samples yielded a 465 base pair product, and sequencing confirmed the presence of a single-peak sequence, validating the F1 generation as CYP2J3^−/−^ (Figure S1).

### 3.4. Genotype Testing of Breeding Offspring

PCR analysis with two primers distinguished between pure and heterozygous zygotic rats. CYP2J3^−/−^ pure rats 465 bp, CYP2J3^+/−^ heterozygous rats of 465 bp and 822 bp, and wild-type rats 822 bp (Figure S2).

### 3.5. Effect of CWP on Kidney Injury Indices Following Acute Heat Stress in Wild-Type and CYP2J3^−/−^ Rats

**Figure 2:**
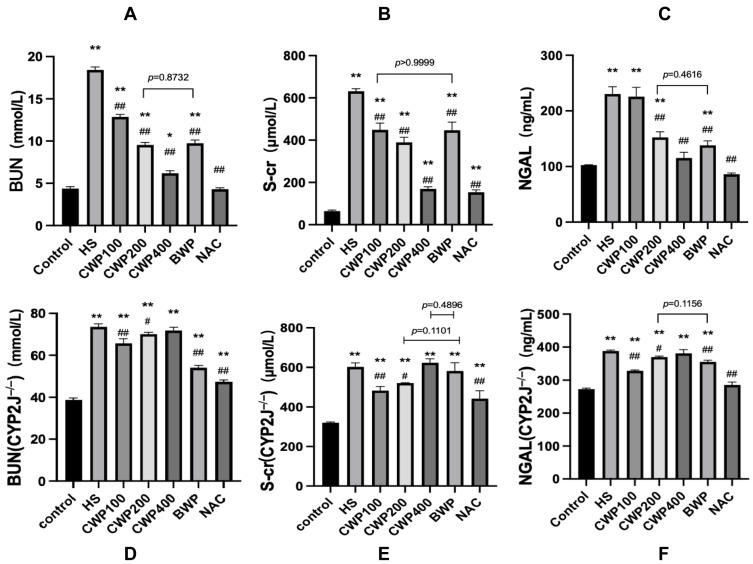
Assessment of kidney injury markers in wild-type and CYP2J3^−/−^ rats. Panels (**A**–**C**) depict the levels of blood urea nitrogen (BUN), serum creatinine (S-cr), and neutrophil gelatinase-associated lipocalin (NGAL) in wild-type rats. Panels (**D**–**F**) present the corresponding kidney injury markers for CYP2J3^−/−^ rats under identical experimental conditions. The treatment groups include: control group (saline gavage, 1 mL/day for 10 days), HS group (saline gavage, 1 mL/day for 10 days), CWP100 group (CWP gavage, 100 mg/kg for 10 days), CWP200 group (CWP gavage, 200 mg/kg for 10 days), CWP400 group (CWP gavage, 400 mg/kg for 10 days), BWP400 group (BWP gavage, 400 mg/kg for 10 days), and NAC group (intraperitoneal injection of N-acetylcysteine, 100 mg/kg for 10 days). All groups, except the control, were subjected to acute heat stress treatment for 3 h, followed by a 9 h rewarming period. Data are expressed as mean ± SEM, based on six animals per group across the seven groups. “*” indicates a significant difference compared to the control group (*p* < 0.05), and “**” indicates a highly significant difference compared to the control group (*p* < 0.01); “#” indicates a significant difference compared with the HS group (*p* < 0.05) and “##” indicates a highly significant difference compared with the HS group (*p* < 0.01).

BUN, serum creatinine (S-cr), and NGAL are established markers of kidney injury. Figure 2A–C illustrate the assessment of these kidney injury markers in wild-type rats following 3 h of acute heat stress and 9 h of recovery at room temperature. Figure 2D–F display the corresponding data for CYP2J3^−/−^ rats under the same conditions.

In the context of the BUN index, the concentration of BUN in wild-type rats (Figure 2A) decreased in a dose-dependent manner following CWP intervention. In contrast, the BUN levels in CYP2J3^−/−^ rats (Figure 2D) progressively approached those of the HS group as the CWP dosage increased, indicating that the knockout of the *CYP2J3* gene exacerbates kidney injury by increasing the metabolic burden.

For the S-cr index, wild-type rats (Figure 2B) also exhibited a dose-dependent decrease in levels following CWP treatment, while CYP2J3^−/−^ rats (Figure 2E) displayed an opposing trend, showing no alleviation of kidney damage.

In the NGAL index, wild-type rats (Figure 2C) exhibited no significant relief effect following administration of CWP at a low dose of 100 mg/kg. However, NGAL content was significantly lower than that in the HS group after treatment with a medium dose of 200 mg/kg. At a high dose of 400 mg/kg, there was no significant difference in NGAL content compared to the control group. In contrast, CYP2J3^−/−^ rats (Figure 2F) did not demonstrate any significant trend of improvement following CWP intervention, with NGAL levels remaining significantly higher than those of the control group after treatment with the high dose of 400 mg/kg.

### 3.6. Effect of CWP on Pathological and Histological Changes in the Kidney of Wild-Type and CYP2J3^−/−^ Rats After Acute Heat Stress

**Figure 3:**
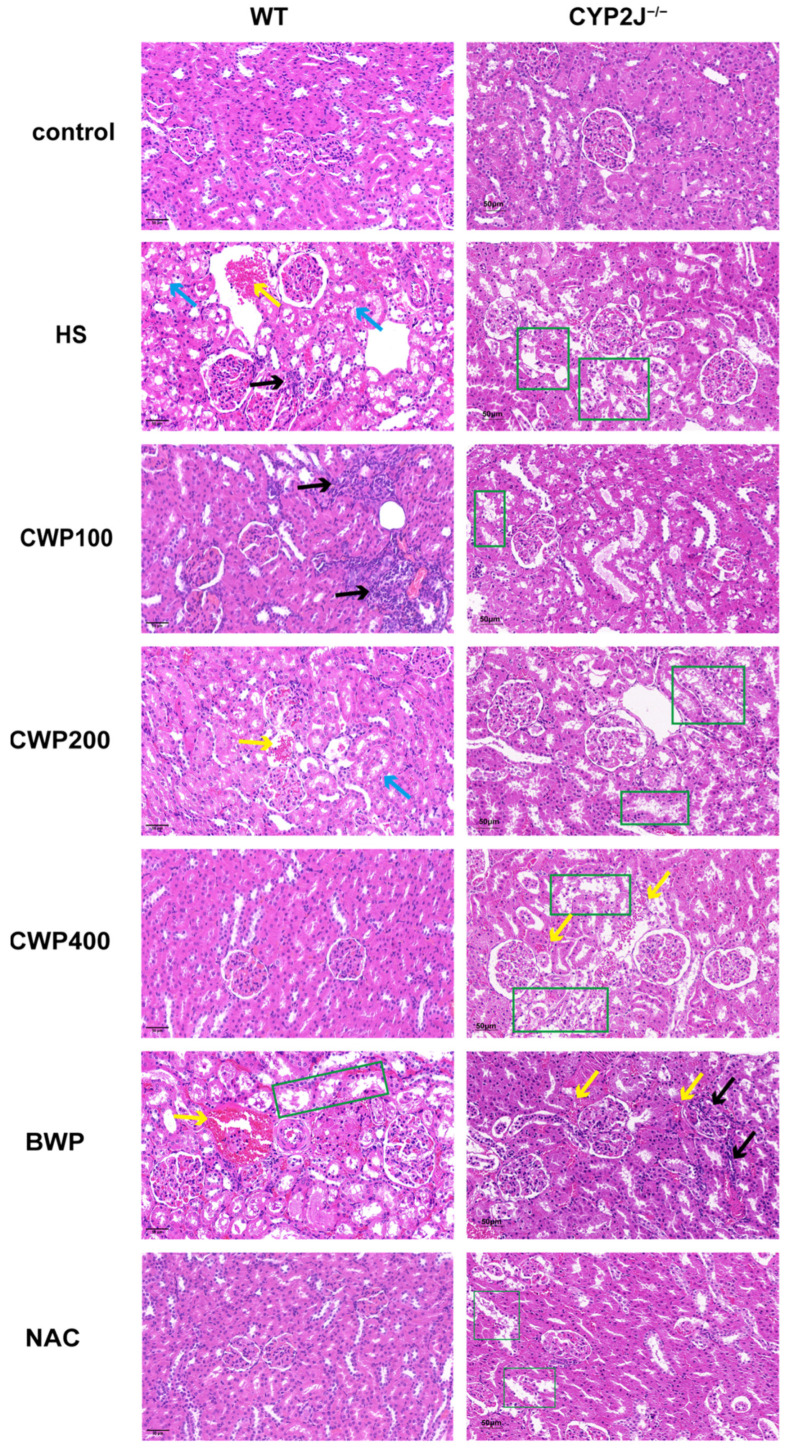
Histopathological changes in the kidneys of wild-type and CYP2J3^−/−^ rats following acute heat stress (H&E staining, 200× magnification, *n* = 6). Kidney tissues were collected for hematoxylin and eosin (H&E) staining, and the displayed images represent a field of view at 200× magnification. The left side shows pathological histology for various groups of wild-type rats, while the right side presents the histology for CYP2J3^−/−^ rats. Yellow arrows indicate areas of renal hemorrhage, black arrows denote inflammatory cell infiltration, and blue arrows highlight cell vesicle degeneration, as well as the loss of the internal brush border structure within the region marked by the green box.

Figure 3 presents a comparative analysis of histopathological changes in the kidneys of wild-type and CYP2J^−/−^ rats following acute heat stress. The control group demonstrated overall structural integrity and full cellular morphology. Wild-type rats in the HS group exhibited hemorrhage, inflammatory cell infiltration, and cellular blister degeneration. CYP2J^−/−^ rats displayed disruption of the tissue structure and loss of the internal brush border structure. The wild-type rats in the CWP-intervened group exhibited substantial inflammatory cell infiltration at low doses, hemorrhage, and cellular blister degeneration at medium doses and overall structural integrity and full cellular morphology at high doses. The CYP2J^−/−^ rats in the CWP-intervened group demonstrated a range of histological disruptions and the disappearance of the internal brush border structure. Additionally, a small amount of hemorrhage was observed at the highest doses. The wild-type rats in the BWP group exhibited hemorrhage and loss of the internal brush border structure, while the CYP2J^−/−^ rats displayed hemorrhage and inflammatory infiltration. The wild-type rats in the NAC group demonstrated overall structural integrity and full cellular morphology, whereas the CYP2J^−/−^ rats exhibited partial structural disruption. The overall comparison revealed that the wild-type rats that received the CWP intervention at high doses exhibited a more robust protective effect on the kidneys, whereas the CYP2J^−/−^ rats that received the CWP intervention did not demonstrate a notable protective effect.

### 3.7. CWP Can Inhibit Kidney Oxidative Stress Through CYP2J and Enhance the Ability of Antioxidant Defense

**Figure 4:**
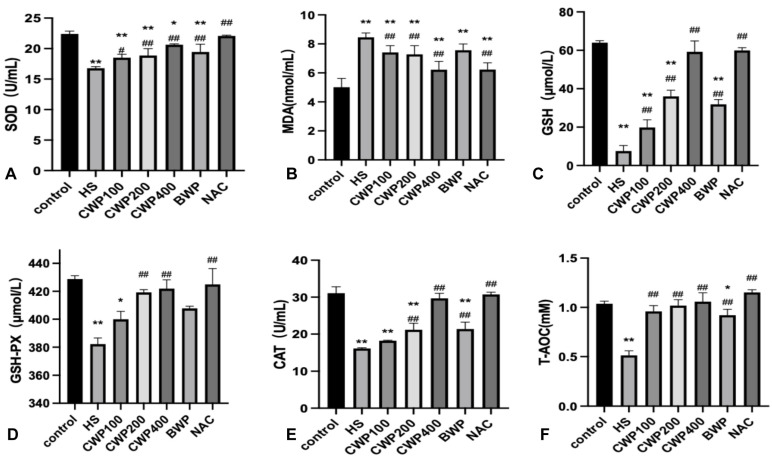
Effect of CWP on renal oxidative stress indices in wild-type rats after acute heat stress. (**A**) Superoxide dismutase (SOD) activity, (**B**) Malondialdehyde (MDA) level, (**C**) Glutathione (GSH) level, (**D**) Glutathione peroxidase (GSH-PX) activity, (**E**) catalase (CAT) level, (**F**) Total antioxidant capacity (T-AOC). The treatment groups include: control group (saline gavage, 1 mL/day for 10 days), HS group (saline gavage, 1 mL/day for 10 days), CWP100 group (CWP gavage, 100 mg/kg for 10 days), CWP200 group (CWP gavage, 200 mg/kg for 10 days), CWP400 group (CWP gavage, 400 mg/kg for 10 days), BWP400 group (BWP gavage, 400 mg/kg for 10 days), and NAC group (intraperitoneal injection of N-acetylcysteine, 100 mg/kg for 10 days). All groups, except the control, were subjected to acute heat stress treatment for 3 h, followed by a 9-h rewarming period. Data are expressed as mean ± SEM, based on six animals per group across the seven groups. “*” indicates a significant difference compared to the control group (*p* < 0.05), and “**” indicates a highly significant difference compared to the control group (*p* < 0.01); “#” indicates a significant difference compared with the HS group (*p* < 0.05) and “##” indicates a highly significant difference compared with the HS group (*p* < 0.01).

**Figure 5:**
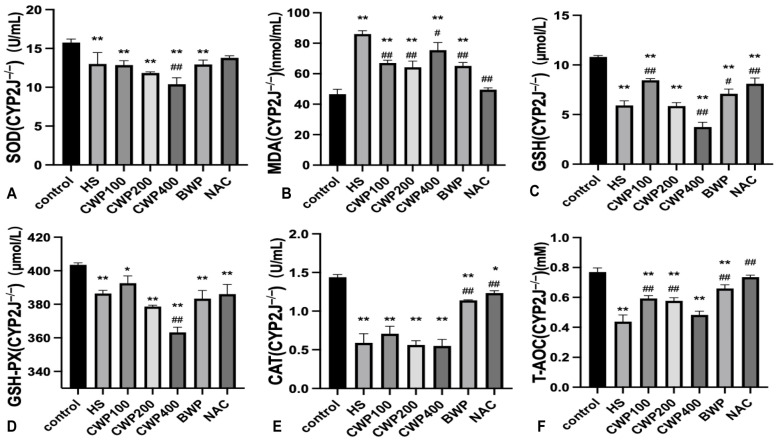
Effect of CWP on renal oxidative stress indices in CYP2J3^−/−^ rats after acute heat stress. (**A**) Superoxide dismutase (SOD) activity, (**B**) Malondialdehyde (MDA) level, (**C**) Glutathione (GSH) level, (**D**) Glutathione peroxidase (GSH-PX) activity, (**E**) catalase (CAT) level, (**F**) Total antioxidant capacity (T-AOC). The treatment groups include: control group (saline gavage, 1 mL/day for 10 days), HS group (saline gavage, 1 mL/day for 10 days), CWP100 group (CWP gavage, 100 mg/kg for 10 days), CWP200 group (CWP gavage, 200 mg/kg for 10 days), CWP400 group (CWP gavage, 400 mg/kg for 10 days), BWP400 group (BWP gavage, 400 mg/kg for 10 days), and NAC group (intraperitoneal injection of N-acetylcysteine, 100 mg/kg for 10 days). All groups, except the control, were subjected to acute heat stress treatment for 3 h, followed by a 9 h rewarming period. Data are expressed as mean ± SEM, based on six animals per group across the seven groups. “*” indicates a significant difference compared to the control group (*p* < 0.05), and “**” indicates a highly significant difference compared to the control group (*p* < 0.01); “#” indicates a significant difference compared with the HS group (*p* < 0.05) and “##” indicates a highly significant difference compared with the HS group (*p* < 0.01).

Superoxide dismutase (SOD) is a crucial enzyme that maintains the balance between oxidative and antioxidative processes. Following acute heat stress, we observed a significant decrease in SOD activity in the heat stress (HS) group (*p* < 0.01). In wild-type rats, SOD activity gradually recovered with increasing doses of CWP (Figure 4A); however, CYP2J3^−/−^ rats did not exhibit recovery following CWP intervention (Figure 5A).

Malondialdehyde (MDA), a well-established marker of lipid peroxidation, significantly increased in the HS model group (*p* < 0.01). Wild-type rats showed a dose-dependent recovery towards control levels (Figure 4B), whereas MDA levels in the high-dose CWP group of CYP2J3^−/−^ rats increased and remained elevated, approaching those of the HS group (Figure 5B).

Both glutathione (GSH) and glutathione peroxidase (GSH-PX) levels in wild-type rats decreased significantly after acute heat stress (*p* < 0.01). Notably, recovery was pronounced after CWP intervention, particularly in the high-dose CWP400 group, which showed no significant difference compared to the control group (Figure 4C,D). Conversely, GSH and GSH-PX levels in CYP2J3^−/−^ rats decreased significantly following CWP intervention, indicating that CWP not only failed to alleviate oxidative stress in these rats but also disrupted normal GSH and GSH-PX synthesis (Figure 5C,D).

After CWP intervention in wild-type rats, catalase (CAT) levels in the low-dose group did not significantly exceed those in the HS group; however, CAT levels were significantly higher in both the middle and high-dose groups (Figure 4E). In contrast, no significant changes were observed in CAT levels in CYP2J3^−/−^ rats post-CWP intervention (Figure 5E). The T-AOC of wild-type rats clearly recovered after the intervention of CWP, BWP, and NAC (Figure 4F). There was no significant difference between the NAC group and the control group of CYP2J3^−/−^ rats, and there was no significant difference between the high-dose CWP group and the HS group (Figure 5F).

These findings further indicate that CWP intervention effectively mitigates oxidative stress induced by acute heat stress in wild-type rats, but that it has no beneficial effect on CYP2J3^−/−^ rats.

### 3.8. Detection of Kidney Injury in CYP2J3^−/−^ Rats by Immunohistochemistry

**Figure 6:**
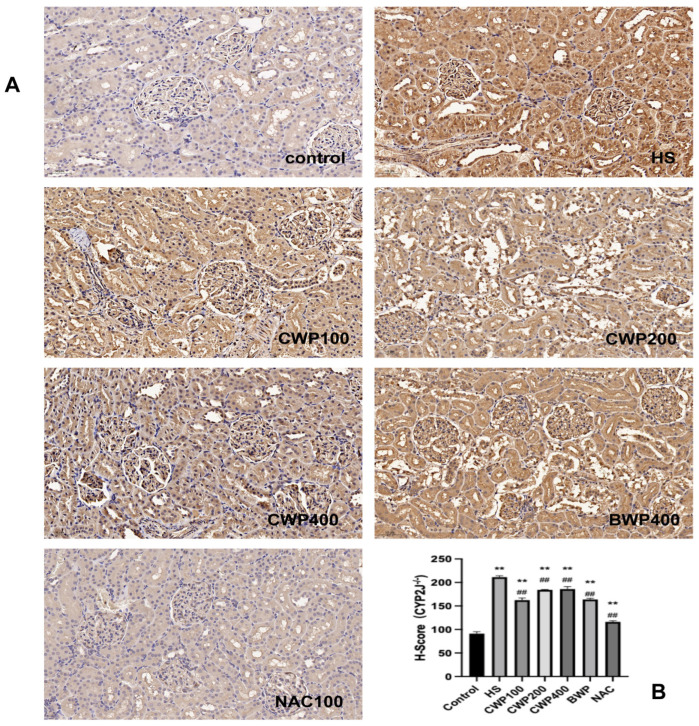
Role of CWP on renal injury in acutely heat-stressed CYP2J3^−/−^ rats detected by immunohistochemical images and Immunohistochemical scores (IHC, 200×, *n* = 6). (**A**) immunohistochemical images, (**B**) Immunohistochemical scores. The treatment groups include: control group (saline gavage, 1 mL/day for 10 days), HS group (saline gavage, 1 mL/day for 10 days), CWP100 group (CWP gavage, 100 mg/kg for 10 days), CWP200 group (CWP gavage, 200 mg/kg for 10 days), CWP400 group (CWP gavage, 400 mg/kg for 10 days), BWP400 group (BWP gavage, 400 mg/kg for 10 days), and NAC group (intraperitoneal injection of N-acetylcysteine, 100 mg/kg for 10 days). All groups, except the control, were subjected to acute heat stress treatment for 3 h, followed by a 9 h rewarming period. Data are expressed as mean ± SEM, based on six animals per group across the seven groups. “**” indicates a highly significant difference compared to the control group (*p* < 0.01), and “##” indicates a highly significant difference compared with the HS group (*p* < 0.01).

Immunohistochemistry and histological scoring (Figure 6) revealed minimal positivity in the control group. In contrast, the HS model group showed increased positivity, characterized by brownish staining and slight inflammatory infiltration. The CWP100 low-dose group exhibited strong positivity around the glomeruli, while the medium- and high-dose groups showed strong positivity in both glomeruli and tubules. There was stronger glomerular positivity in the CWP high-dose group. The NAC-positive control group had weak positivity (Figure 6A). The CWP intervention given remained highly significantly higher than the control group, with no trend towards recovery (Figure 6B). CWP administration following CYP2J gene knockdown did not demonstrate favorable renoprotective effects and may exacerbate renal injury at higher doses, suggesting that CYP2J plays a role in regulating CWP effects on kidneys under acute heat stress.

### 3.9. Effect of CWP on Apoptosis in Kidney Cells of Acutely Heat-Stressed CYP2J3^−/−^ Rats

**Figure 7:**
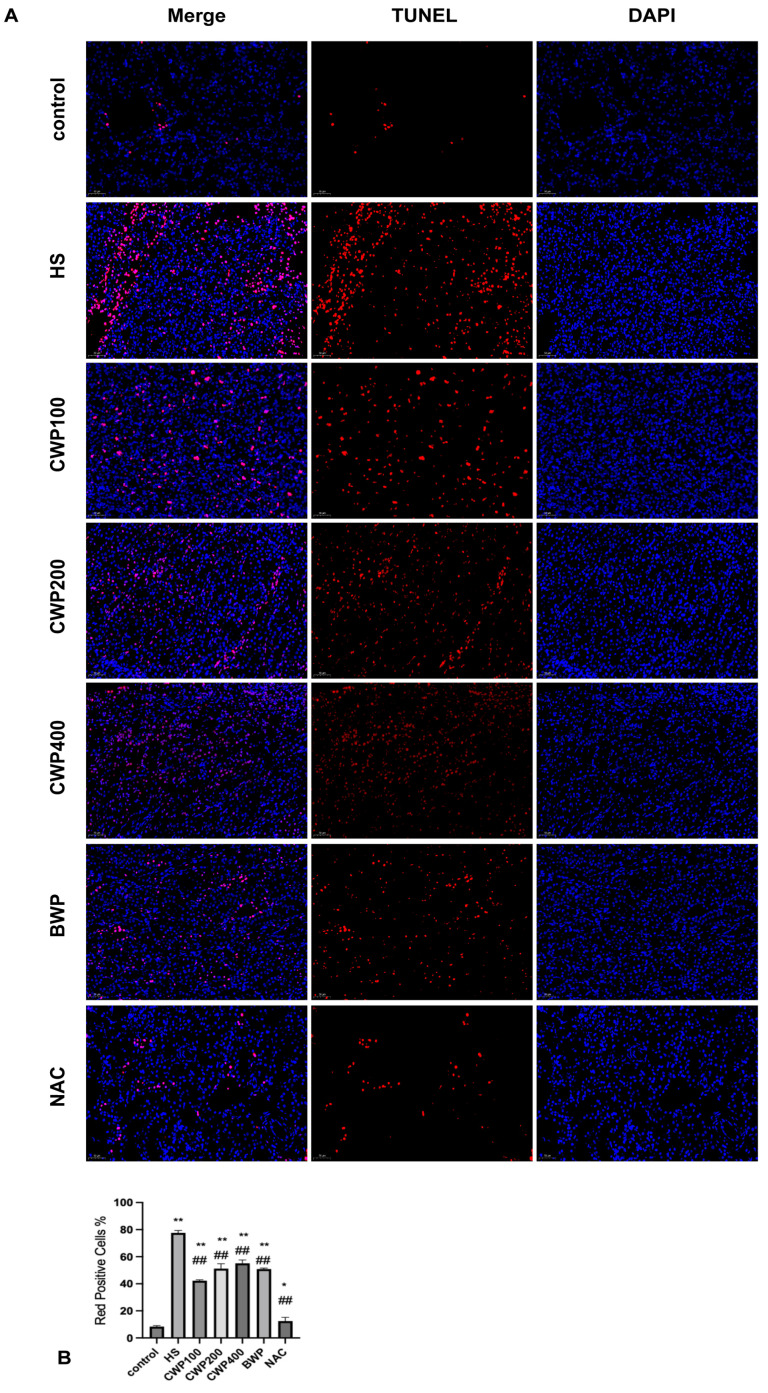
Apoptosis and apoptosis rate in rat kidney cells after acute heat stress (TUNEL, 200×, *n* = 6). (**A**) TUNEL assay to detect apoptosis in acute heat stress rat kidney cells under CWP intervention, 200× field of view, DAPI re-stained nuclei are blue under UV and CY3 fluorescein labelled positive apoptotic nuclei are red. (**B**) Apoptosis rate (red positive cells %). Red light positive rate = total number of red light positive cells/total number of cells. The treatment groups include: control group (saline gavage, 1 mL/day for 10 days), HS group (saline gavage, 1 mL/day for 10 days), CWP100 group (CWP gavage, 100 mg/kg for 10 days), CWP200 group (CWP gavage, 200 mg/kg for 10 days), CWP400 group (CWP gavage, 400 mg/kg for 10 days), BWP400 group (BWP gavage, 400 mg/kg for 10 days), and NAC group (intraperitoneal injection of N-acetylcysteine, 100 mg/kg for 10 days). All groups, except the control, were subjected to acute heat stress treatment for 3 h, followed by a 9 h rewarming period. Data are expressed as mean ± SEM, based on six animals per group across the seven groups. “*” indicates a significant difference compared to the control group (*p* < 0.05), and “**” indicates a highly significant difference compared to the control group (*p* < 0.01), and “##” indicates a highly significant difference compared with the HS group (*p* < 0.01).

To further investigate CWP’s effect on apoptosis in acutely heat-stressed CYP2J3^−/−^ rat kidneys, the TUNEL method was used (Figure 7). After a 3-h acute heat stress period, the HS model group showed a significant increase in apoptosis-positive cells, with enhanced fluorescence intensity and broader distribution compared to the control group. The CWP100 low-dose group had fewer positive cells, though the fluorescence intensity remained high. In the CWP medium- and high-dose groups, the fluorescence intensity was reduced, but the apoptotic cell distribution was more extensive. The NAC-positive control group had more pronounced apoptotic cell fluorescence but fewer positive cells compared to the control group, showing a significant difference from other experimental groups. This indicates that CYP2J3 gene knockdown has a greater impact on heat stress-induced apoptosis and CWP intervention does not provide protective effects.

### 3.10. CWP Activates the PI3K/AKT Signalling Pathway by Regulating CYP2J Activity to Alleviate Renal Injury in HS Rats

**Figure 8:**
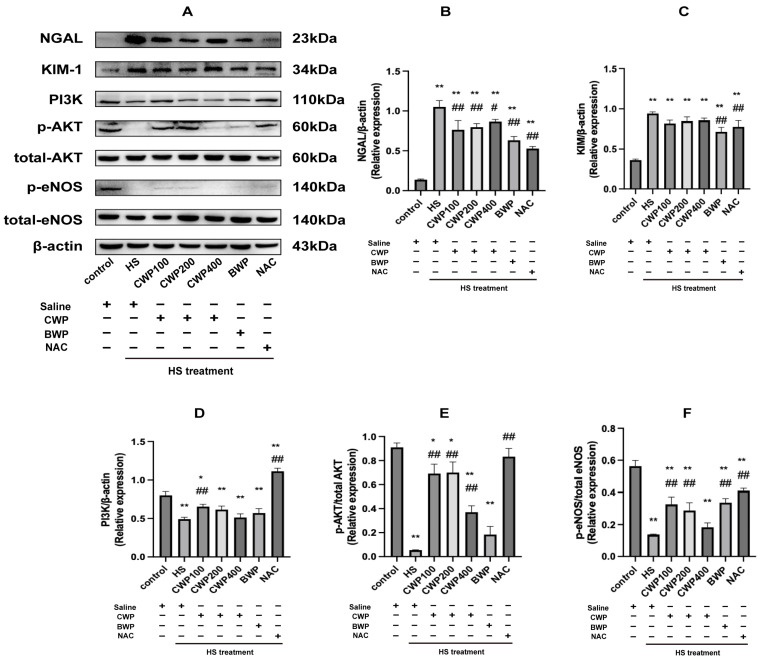
Protein expression of NGAL, KIM-1, and components of the PI3K/AKT/eNOS signaling pathway, indicators of renal injury under acute heat stress in CYP2J3^−/−^ rats. (**A**) shows the Western blotting electrophoresis of protein bands for NGAL, KIM-1, and the PI3K/AKT/eNOS signaling pathways in CYP2J3^−/−^ rats, with β-actin serving as an internal reference. (**B**) illustrates the relative expression of NGAL protein. (**C**) displays the relative expression of KIM-1 protein. (**D**) depicts the relative expression of PI3K protein. (**E**) presents the relative total expression of p-AKT protein, while (**F**) shows the relative total expression of p-eNOS protein. Data are expressed as mean ± SEM, based on six animals per group across the seven groups. “*” indicates a significant difference compared to the control group (*p* < 0.05), and “**” indicates a highly significant difference compared to the control group (*p* < 0.01); “#” indicates a significant difference compared with the HS group (*p* < 0.05) and “##” indicates a highly significant difference compared with the HS group (*p* < 0.01).

To further investigate if CWP activates the PI3K/AKT pathway via CYP2J and mitigates HS-induced renal injury. Relevant protein expressions were analyzed in CYP2J3^−/−^ rats. Figure 8A presents the relative expression data, with Figure 8B,C depicting NGAL and KIM-1 levels, respectively—markers of kidney injury. Post-HS, the HS model group exhibited significantly elevated expression, consistent with ELISA results. However, CWP intervention did not lead to noticeable improvements, particularly in KIM-1 levels, with no significant difference between the CWP and HS groups. These results suggest that CWP does not offer protective effects against kidney injury in CYP2J3^−/−^ rats.

PI3K expression was significantly reduced in the HS group compared to controls and was further suppressed under stress conditions. In contrast, the CWP100 low-dose group showed increased PI3K expression, while the middle and high-dose CWP groups, along with the BWP group, exhibited suppressed PI3K levels. Activation of PI3K was observed only in the NAC-positive control group (Figure 8D). Apart from the NAC-positive group, which showed activated p-AKT relative to total AKT, all other groups exhibited significant inhibition of p-AKT. The CWP400 high-dose group demonstrated comparable PI3K expression to the HS model group, with more pronounced inhibition (Figure 8E). p-NOS levels were similarly repressed relative to total NOS across all the experimental groups (Figure 8F).

In summary, CWP intervention following CYP2J3 gene knockdown did not activate the PI3K/AKT pathway or mitigate renal injury in CYP2J3^−/−^ rats.

## 4. Discussion

Camel milk contains trace amounts of arachidonic acid [27], which can be metabolized to produce eicosatrienoic acids (EETs) through the action of cytochrome P450 2J (CYP2J) [28,29]. α-Lactalbumin, a protein enriched in CWP, is involved in the synthesis of AA. Given this, investigating the interaction between CWP and CYP2J is crucial. Our findings indicate that CWP enhances CYP2J activity, thereby facilitating EET synthesis [13,30].

To further confirm that the activation of the PI3K/AKT pathway by CWP operates via CYP2J, the present study was conducted using CYP2J3^−/−^ rats. It was observed that following the knockout of the CYP2J3 gene, the renal damage sustained by rats subjected to HS was more pronounced, with the highest dose of CWP resulting in a more severe injury than the lower to medium doses. There is a paucity of literature indicating that the knockout of the CYP2J3 gene directly affects renal function in rats. However, there is a report suggesting that renal dysfunction can influence protein metabolism within the organism [31,32,33]. The primary products of protein metabolism in the body are BUN (blood urea nitrogen) and S-cr (serum creatinine). If renal function is affected, there will be an accumulation of BUN and S-cr in the body, which will further exacerbate the burden on the kidneys [34]. In this instance, the considerable quantity of protein consumed may exert additional pressure on the kidneys, and in conjunction with the stimulation of HS, render them more susceptible, thereby exacerbating the extent of damage [35]. The pathological section results demonstrate that the kidney, following HS, exhibited not only cell degeneration and rupture, but also damage to the tissue structure, characterized by the presence of numerous dilated renal tubules, an enlarged official lumen, and a disappearance of the brush border. Furthermore, it was demonstrated that HS exacerbated renal damage following the knockdown of the CYP2J3 gene, and that the administration of CWP was unable to mitigate this effect.

The regulation of CYP2J expression has the potential to influence the treatment and prognosis of numerous diseases. Its role in the intervention of liver disease and tumor treatment [36], as well as the prevention of thrombosis and hypertension, is of particular significance [37]. Furthermore, CYP2J3 and CYP2J2 are highly homologous in the evolutionary tree, which means that the successful construction of CYP2J3^−/−^ rats can not only facilitate the study of the role of PI3K/AKT in antioxidant research, but also provide a robust experimental basis for the prevention of related diseases [14,38].

PI3K/AKT also plays an important role in OS. Arab H H (2018) used CM to activate the PI3K/AKT pathway and inhibit OS, which in turn attenuated 5-fluorouracil-induced renal injury in rats [5]. Arbutin was employed to inhibit heat stress-induced apoptosis through the activation of the PI3K/AKT signaling pathway, thereby promoting the proliferation and migration of heat-injured dermal fibroblasts and keratinocytes [39]. The cytoprotective effect of butein on OS was mediated by the upregulation of manganese superoxide dismutase expression through the PI3K/AKT/Nrf2 pathway, as indicated by Zhang Rui in his study [40]. A number of studies have demonstrated that the PI3K/AKT pathway can inhibit the onset and progression of OS. This study also revealed that the administration of CWP following the knockdown of the CYP2J3 gene did not result in the alleviation of OS indicator expression within the organism. Furthermore, the expression of the PI3K/AKT pathway was also investigated. The knockdown of the CYP2J3 gene was also found to inhibit the activation of the PI3K/AKT pathway, which did not alleviate the damage caused by OS or inhibit apoptosis in a timely manner. Consequently, the validation of apoptosis using TUNEL cells revealed a significant level of apoptosis. Furthermore, the TUNEL apoptosis validation revealed a significant degree of apoptosis, with only NAC-positive drugs exhibiting antioxidant properties and inhibiting the apoptotic process.

## 5. Conclusions

The use of CYP2J3^−/−^ rats revealed that under acute heat stress, the intervention of CWP was unable to activate the PI3K/AKT pathway and act as an antioxidant. In contrast, the NAC-positive drug control group was able to maintain the antioxidant response of the organism. It can therefore be demonstrated that CWP is capable of activating the PI3K/AKT pathway by regulating the activity of CYP2J, thereby mitigating the damage caused by OS.

## Figures and Tables

**Table 1:** Protein enrichment of highly expressed proteins related to antioxidants in CWP and BWP.

NAME	CWP	BWP
Albumin	369	338
Lactotransferrin	174	150
Lactoperoxidase	98	83
α-Lactalbumin	278	240
Superoxide Dismutase	3	4
Heat Shock Protein	12	10
L-Lactic Dehydrogenase	3	2
Cysteine-Rich Secretory Protein	16	17
NADH Reductase	3	5
Peroxiredoxin	1	-
Antioxidant Enzymes (nucleus)	1	-
Glutathione Hydrolase	8	9

## Data Availability

Some of the data supporting the results of this study are available in the Supplementary Material to this article, and other data are available from the corresponding author upon reasonable request.

## Supplementary Materials

The following supporting information can be downloaded at: www.mdpi.com/xxx/s1,s2 Figure S1: CYP2J3 genotype sequence determination and genotyping of the F1 generation. Figure S2: PCR identification of offspring rat genotype.

## Abbreviation

Heat stress (HS); Oxidative stress (OS); cytochrome P450 2J (CYP2J); eicosatrienoic acids (EETs); Camel whey protein (CWP); Bovine whey protein (BWP); Malondialdehyde (MDA); N-Acetylcysteine (NAC); superoxide dismutase (SOD); Glutathione peroxidase (GSH-Px); Catalase (CAT); Urea nitrogen (BUN); Serum creatinine (S-cr); Neutrophil gelatinase-associated lipocalin (NGAL); Total antioxidant capacity (T-AOC).
