# Supplementary material for: Camel Whey Protein Attenuates Acute Heat Stress-Induced Kidney Injury in Rats by Up-Regulating CYP2J Activity and Activating PI3K/AKT/eNOS to Inhibit Oxidative Stress
Source: Vet Sci. 2024 Oct 28;11(11):524. doi: 10.3390/vetsci11110524 (PMC11599098; doi:10.3390/vetsci11110524)
Supplement: Supplementary file 1 [file vetsci-11-00524-s001.zip › vetsci-3194898-supplementary.pdf]

Supplementary Information

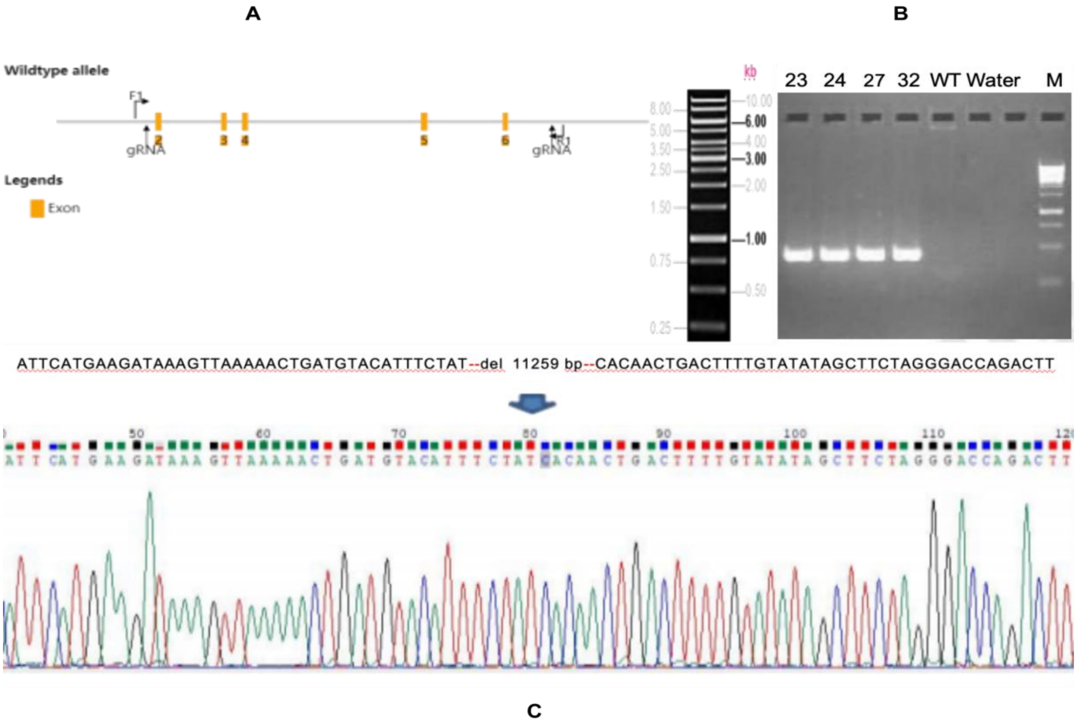

Figure S1. CYP2J3 genotype sequence determination and genotyping of the F1 generation

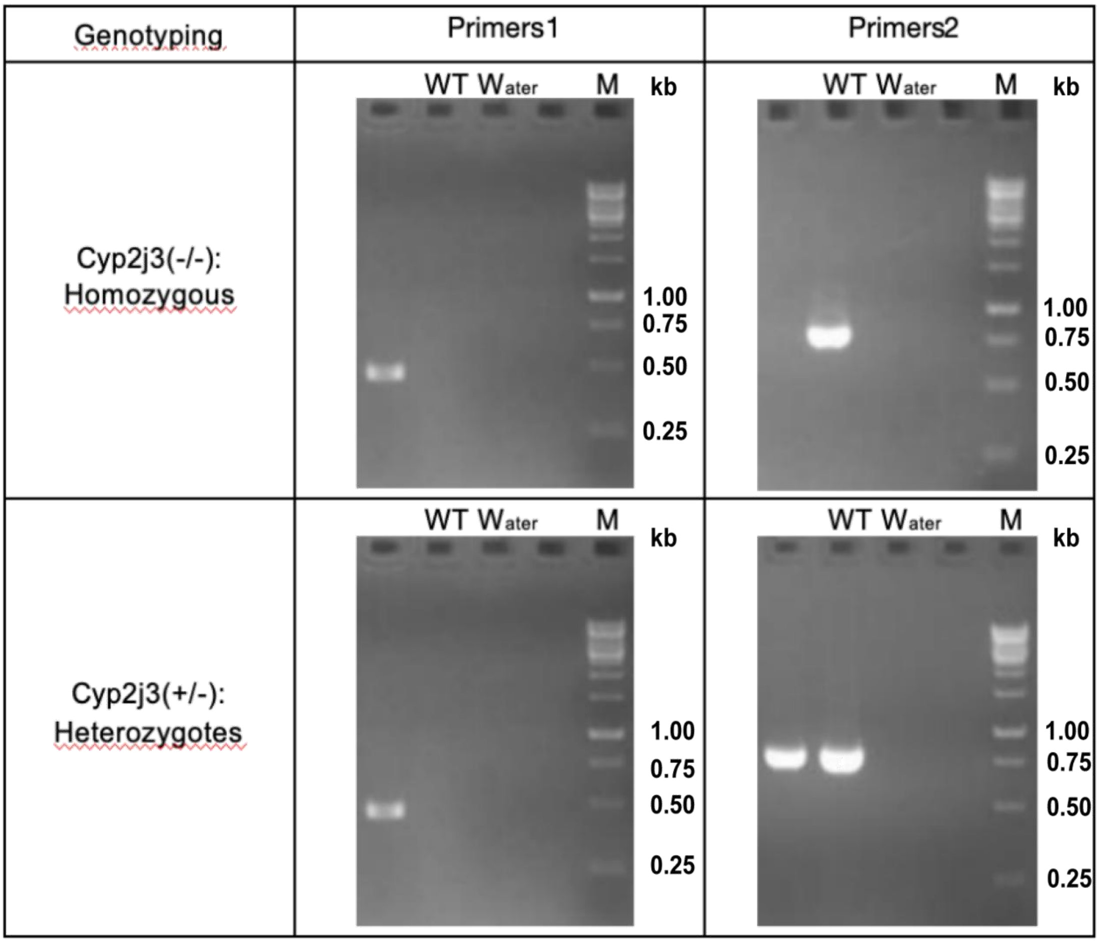

Figure S2. PCR identification of offspring rat genotype
